# Genome-wide detection and analysis of homologous recombination among sequenced strains of *Escherichia coli*

**DOI:** 10.1186/gb-2006-7-5-r44

**Published:** 2006-05-31

**Authors:** Bob Mau, Jeremy D Glasner, Aaron E Darling, Nicole T Perna

**Affiliations:** 1 Department of Mathematics, Lincoln Drive, University of Wisconsin, Madison WI 53706, USA; 2 Department of Oncology, University Ave, University of Wisconsin, Madison WI 53706, USA; 3 Genome Center of Wisconsin, Henry Mall, University of Wisconsin, Madison WI 53706, USA; 4 Department of Computer Science, W. Dayton St, University of Wisconsin, Madison WI 53706, USA; 5 Department of Animal Health and Biomedical Sciences, Linden Drive, University of Wisconsin, Madison WI 53706, USA

## Abstract

**Background:**

Comparisons of complete bacterial genomes reveal evidence of lateral transfer of DNA across otherwise clonally diverging lineages. Some lateral transfer events result in acquisition of novel genomic segments and are easily detected through genome comparison. Other more subtle lateral transfers involve homologous recombination events that result in substitution of alleles within conserved genomic regions. This type of event is observed infrequently among distantly related organisms. It is reported to be more common within species, but the frequency has been difficult to quantify since the sequences under comparison tend to have relatively few polymorphic sites.

**Results:**

Here we report a genome-wide assessment of homologous recombination among a collection of six complete *Escherichia coli *and *Shigella flexneri *genome sequences. We construct a whole-genome multiple alignment and identify clusters of polymorphic sites that exhibit atypical patterns of nucleotide substitution using a random walk-based method. The analysis reveals one large segment (approximately 100 kb) and 186 smaller clusters of single base pair differences that suggest lateral exchange between lineages. These clusters include portions of 10% of the 3,100 genes conserved in six genomes. Statistical analysis of the functional roles of these genes reveals that several classes of genes are over-represented, including those involved in recombination, transport and motility.

**Conclusion:**

We demonstrate that intraspecific recombination in *E. coli *is much more common than previously appreciated and may show a bias for certain types of genes. The described method provides high-specificity, conservative inference of past recombination events.

## Abstract 2

Multiple alignment of *E. coli *and *Shigella *genomes reveals that intraspecific recombination is more common than previously thought.

## Background

The role of lateral gene transfer (LGT) in shaping prokaryotic genomes has been the subject of intense investigation and debate in recent years [1-10]. In the pre-genomic era, the handful of examples of LGT were detected primarily as discordance between phylogenetic reconstructions with different housekeeping genes [11-14]. The explosion of publicly available bacterial genome sequences, coupled with the development of whole-genome comparison tools [15-17], initially focused LGT discovery on genome-wide scans for islands of sequences specific to particular lineages of bacteria (for example, [18-21]). Most recently, phylogenetic approaches are applied to detect LGT among genome-wide sets of putative orthologs [2,9,10]. Together, these studies point to low, but detectable, levels of LGT among distantly related species with occasionally higher rates found among organisms that occupy similar environments. Closely related organisms show higher levels of LGT, with intraspecific comparisons showing the highest levels. Two limitations of these analyses are the lack of phylogenetic resolution, particularly among intraspecific comparisons, and the reliance on annotated boundaries of genes in delineating candidate regions.

Statistical and phylogenetic methods have been developed for detecting recombination in aligned sequences of single genes or relatively short genomic segments. One general approach, referred to as nucleotide substitution distribution methods in [22], assesses atypical clusters of nucleotide differences. Clusters come in two flavors: groups of polymorphisms exhibiting the same topologically discordant pattern [23,24], or an elevated rate of mutation in a single lineage across a segment of the alignment [25-28]. The former indicates recombination between compared strains, while the latter implies a recombination with some unknown, more divergent, strain. Phylogenetic methods are most often applied in the context of detecting recombination break points in sequence alignments [29-32]. These methods require longer alignments, are computationally intensive, and have reportedly been outperformed by substitution distribution methods on simulated test data [33].

Genome-scale analyses of lateral transfer events have typically relied on identification of incongruent tree topologies from phylogenetic analyses of sets of putative orthologous genes identified by reciprocal BLAST analyses [7,9,34]. This approach can be confounded by errors associated with BLAST, such as false-positive orthologs, is limited to identifying recombination events that occur within gene boundaries, and is unlikely to identify short recombined regions within genes.

Recently, a Markov clustering algorithm was used to partition orthologous pairs of genes, determined by an all versus all BLAST comparison of 144 fully sequenced prokaryotic genomes, into maximally representative clusters [10,35]. Bayesian phylogenetic analysis (for example, [36,37]) was applied to each cluster of four or more taxa to infer lateral gene transfer against the background of a consensus 'supertree' of sequenced bacteria. This approach is most successful in determining global pathways of gene transfer between phyla and divisions of prokaryotes, where homologous recombination is unlikely to have played a significant role. Rather, these likely arise as illegitimate recombination events.

Here, we develop a method to detect segments of closely related genomes that have been replaced with a homologous copy from another conspecific lineage, that is, an allelic substitution. The method is not designed to detect non-homologous sequences that may have accompanied a homologous recombination event or homologous recombination events involving identical alleles.

The method compiles a list of polymorphism sites from a whole-genome multiple alignment, then applies score functions to locate clusters discordant with the predominant phylogenetic signal. Identified clusters can cross gene boundaries and non-coding sequence. Our use of extreme value theory furnishes us with a statistically defensible criterion to assess significance of these clusters in much the same manner as the Karlin-Altschul statistics help interpret BLAST results [38,39].

We apply the recombination detection method to the published genome sequences of several *E. coli *[18,40-44]. Construction of a multiple whole genome alignment facilitates a global survey of recombination among these *E. coli *isolates. Genome sequences must first be partitioned into locally collinear blocks (LCBs) - regions without rearrangement. Most LCBs contain lineage-specific sequence acquired through lateral gene transfer or differential gene loss. To further complicate matters, non-homologous sequences from different organisms can integrate into different lineages at a common locus [18]. In a previous work, we developed a software package called Mauve [17] that can construct global multiple genome alignments in the presence of rearrangement and lineage-specific content. The Mauve alignments provide a convenient starting point for locating polymorphic patterns indicative of intraspecific recombination, which we call allelic substitution.

## Results

**Figure 1:**
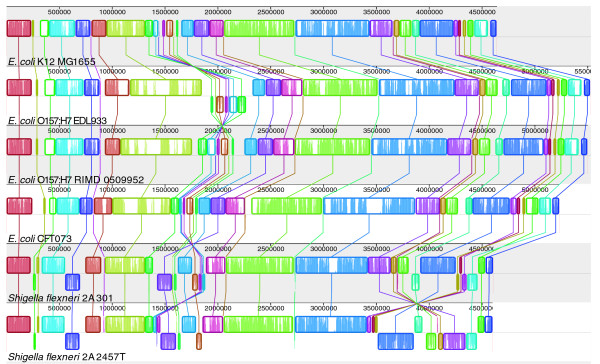
A multiple whole-genome alignment of six strains consists of 34 rearranged pieces larger than 1 kb. Each genome is laid out horizontally with homologous segments (LCBs) outlined as colored rectangles. Regions inverted relative to *E. coli *K-12 are set below those that match in the forward orientation. Lines collate aligned segments between genomes. Average sequence similarities within an LCB, measured in sliding windows, are proportional to the heights of interior colored bars. Large sections of white within blocks and gaps between blocks indicate lineage specific sequence.

As seen in Figure 1, the Mauve genome aligner takes the four *E. coli *and two *Shigella flexneri *genome sequences and returns 34 local alignments spanning 3.4 Mb of homologous sequence common to all strains. The majority of rearrangements occur in *Shigella *genomes where inversions between copies of repetitive elements are relatively frequent [40].

Computer assisted screening of the Mauve output finds 733 problematic intervals inside LCBs in which base pairs do not properly align because of gaps created by lineage specific sequence and/or attempts to align non-homologous sequence. Deleting these intervals from the alignment yields 130,008 high quality base pair differences. Common bipartitions, constituting 96.4% of all such differences, are listed in Table 1.

**Figure 2:**
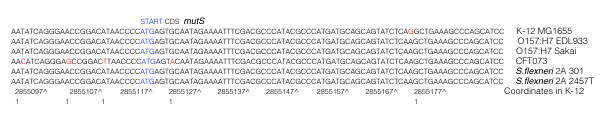
Small sample segment of the alignment spanning the start of the *mutS *gene (denoted in blue). Location of a mismatch is indicated by the integer '1' along the bottom row. Five columns contain SNDs: TTTCTT, AAAGAA, AAATAA, GGGAGG, and GAAAAA. The first four share the same bipartition pattern (111211) and are deemed equivalent, even though one of them results from a transversion. The other SND is considered distinct despite having the same mutation (A to G) found in the second SND.

We use the term 'single nucleotide difference' (SND) to describe the partition structure at a variable site in the alignment. A representative 100 base-pair (bp) segment of the 3.4 Mb alignment is presented in Figure 2 for illustrative purposes.

All but 2% of variable sites are bi-allelic, meaning each site splits six strains into two groups, called a bipartition. Nearly 80% of the bi-allelic SNDs have a minor allele unique to the CFT, K-12, O157:H7, or *S. flexneri *lineage. The remaining bi-allelic SNDs divide the lineages into three alternative pairings of sister taxa, giving rise to three alternative unrooted tree topologies denoted as: ψ_*KS *_(K-12 with *S. flexneri*, CFT with O157:H7); ψ_*KO *_(K-12 with O157:H7, CFT with *S. flexneri*); and ψ_*KC *_(K-12 with CFT, O157:H7 with *S. flexneri*).

The four lineages serve as operational taxonomic units (OTUs) in our study of allelic substitution in *E. coli*. When nucleotides at a polymorphic site exhibit a partition structure explainable by a single point mutation, the induced bipartition is said to be compatible with the enabling topology. Bipartitions labeled KS, KO, and KC in Table 1 are compatible with the topologies ψ_*KS*_, ψ_*KO*_, and ψ_*KC*_, respectively. Note that frequency of the KS pattern exceeds that of each of its competitors by 3,000 SNDs, thus certifying ψ_*KS *_as the 'species' topology. The elevated frequency of SNDs unique to CFT roots topology ψ_*KS *_as (((KS)O)C). The 102,000 topologically uninformative lineage-specific SNDs nevertheless provide information that our method uses to assess recombination.

**Figure 3:**
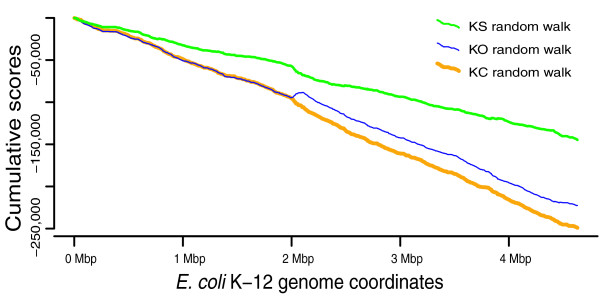
Three excursions (KS, KO, and KC) spanning the alignment with K-12 MG1655 as reference genome. The KS random walk plot, representing the dominant clonal topology, decreases more gradually than do the two other plots. Excursions for the discordant topologies (patterns KO and KC) run parallel to one another, except in a 100 kb region at 2 Mb where KO abruptly increases. Parallel flat gaps common to all three plots reflect K-12 lineage specific sequence.

We define three complementary score functions that discriminate between KS, KO, and KC patterns. Each of these score functions assigns an integer value to each SND pattern. Moving across the chromosome of reference strain MG1655, we keep a cumulative sum of the scores assigned by each function to consecutive SNDs in the alignment. Graphical representations of cumulative scores, called random walk plots or excursions, can reveal large-scale variations in feature composition. Excursions for each of the three topologies are plotted concurrently in Figure 3.

A large phylogenetic anomaly appears midway through the alignment. Magnification of a 100 kb segment between 1.95 and 2.1 Mb reveals a core 40 kb region in which KO SNDs are the dominant pattern of substitution, flanked by transitional regions for which ψ_*KO *_serves as the 'gene tree' as well.

Global random walk plots highlight grossly deviant regions. In this alignment, a solitary segment stands out. All other regions appear indistinguishable from one another in Figure 3. Unless stated to the contrary, DNA sequence and genes from the large atypical region (from *sdiA *to *gnd*) are excluded from further computations (a separate analysis of this region is included in Appendix 2 of Additional data file 1).

### Local variation in phylogenetic signal

In Figure 3, clusters of like patterns labeled KS, KC, or KO generate tiny, imperceptible bumps in the corresponding random walk plots. Examined at higher resolution (data not shown), they can be seen to punctuate each excursion. However, manual scanning of high-resolution random walk plots is tedious, time consuming, and error-prone. In Materials and methods, we describe an alternative strategy that automatically scans for clusters at the local level.

The score functions generating Figure 3 are designed to elicit large positive local scores (differences in cumulative scores evaluated at nearby positions) whenever clusters of like, topologically informative, patterns are encountered. When that local score exceeds a predetermined threshold, the interval between the delimiting SNDs is declared a high scoring segment (HSS). The strategy behind this scheme is exactly analogous to BLAST [38], in which high scoring segments denote probable homology between the query and one or more reference sequences.

When two lineages share a nucleotide that is not the result of a single mutation in a common ancestor, a homoplasy is said to have occurred. Homoplasies arise either through multiple mutations at a common site (convergent evolution) or recombination. The former tend to be distributed randomly about an alignment, whereas a recombination event typically produces a cluster of nucleotide differences at nearby sites exhibiting the same SND pattern. Our approach identifies such clusters of nucleotide differences with a common phylogenetic partitioning pattern. Variability in mutation rates and patterns in different chromosomal regions and bacterial lineages might also lead to physical clustering of similar substitutions. Although the clustering of sites with similar patterns strongly suggests homologous recombination between lineages, we cannot rule out the possibility that some clusters arise by independent mutation-driven processes. Simple score functions alone cannot distinguish between these two possibilities, though the latter is believed to be relatively rare.

Our method relies on the relative intensity of particular SND patterns (the one of interest versus all others) to measure cluster formation, rather than the absolute number of SNDs in any given fixed length segment of the alignment. As a result, local mutational intensity is factored out of the analysis. We assert this is legitimate provided the overall rate of mutation is not too great, and local deviations from that average are not severe. We demonstrate in appendix 5 of Additional data file 1 that this is indeed the case for these six genomes. Random SNDs can and do form clusters of identical patterns simply by chance. Given the number of SNDs and their relative frequencies within the alignment, we wish to distinguish 'bumps' that are too large to have occurred by chance.

Here again, BLAST statistics [39] serve as the model for assessing significance. Random walk theory provides the tools for assessing high scoring segments, and the corresponding extreme value distributions (EVDs) guide selection of appropriate thresholds. Random walks (as opposed to random walk plots) are stochastic processes operating under a fixed set of probabilities at each stage.

In the Materials and methods section, we apply the relevant theory to derive thresholds. Using the appropriate extreme value distribution as an arbiter, we chose a significance threshold of 170 for clusters of KS SNDs and the same value of 100 for both KO and KC, as their frequencies are nearly identical outside the large atypical region (4.85% versus 4.57%). These thresholds define 186 high scoring segments that span 7.5% of the sequence alignment. A breakdown by pattern and range of scores is arrayed in Tables 2 and 3.

We deviate from BLAST protocols in one important respect: a high scoring segment maximizes the local score, which is the primary goal of sequence alignment. Here, we want to isolate sub-regions within an HSS that individually exceed the significance threshold. Our rationale is that sequence between sub-regions may not have participated in the recombination, and we want to identify only those genomic intervals that possess *prima facie *evidence of recombination.

A minimal significant cluster (MSC) is a smallest subset of contiguous SNDs generating a local score above the threshold. To avoid ambiguity, overlapping MSCs supporting the same topology are merged into a single representative MSC. Most high scoring segments consist of a single such cluster, but HSSs with more than 150 SNDs often contain two or more disjoint MSCs.

**Figure 4:**
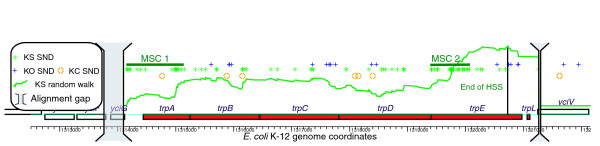
The KS local random walk plot showing homologous recombination in the tryptophan (*trp*) operon. Genes are rectangular boxes positioned above or below the axis based on transcribed strand. KS SNDs form two non-overlapping MSCs with significant local scores exceeding 170. Both MSCs, with a combined length under 2 kb, are contained in a single 6.5 kb HSS covering most the *trp *operon. The positions of each KO, KC, and KS SND in *E. coli *K-12 are shown above the KS excursion. Random walk values below 50 are not plotted, resulting in the absence of visible KC or KO excursions.

HSSs and MSCs are represented graphically by modifying global random walk plots. By subtracting off the underlying negative trend, only positive local scores are displayed. Figure 4 shows a local random walk plot for the HSS covering the seven genes of the tryptophan operon. The *trp *operon was the first reported example of homologous recombination in *E. coli *[45].

Although the entire *trp *operon may have been exchanged in a single event, only *trpA *and *trpE *contain clusters of KS SNDs that individually give rise to statistically significant local scores. Moreover, the first MSC clearly includes in excess of 200 bp downstream of the *trp *operon - evidence that downstream transcription termination signals have also been subject to homologous recombination. In this manner, MSCs facilitate more precise targeting of chromosomal regions implicated in recombination. This criterion modestly increases the number of recombined segments to 216 (75, 62, 79 for KO, KC, KS, respectively) while reducing the amount of participating sequence from 251 kb to 129 kb. We outline a procedure for finding non-overlapping minimal significant clusters inside high scoring segments in Materials and methods.

### Gene content of regions that underwent recent allelic substitution

Although our method identifies recombination events independently of gene boundaries, it is interesting to look at the types of genes and gene products involved in these events. To this end, we extracted a list of genes encoded in regions deemed atypical by our random walks. Among the 4,353 genes in K-12, 3,107 align across all six genomes. Of these, 271 genes intersect a minimal cluster segment. When augmented with 40 genes from the atypical region, 10% of shared genes exhibit evidence of recombination. A table of the 186 high scoring segments, subdivided into MSCs and identifying affected genes, is provided as Additional data file 2.

We examined this list of 311 genes in light of gene function assignments made using a controlled vocabulary called MultiFun [46] that supports multiple functional classifications for a given gene. The 3,107 genes aligned by Mauve in all six genomes have been classified with 5,550 gene functions. Nearly 2,000 genes have a single classification (many are 'Unknown function'). By contrast, six genes have seven 'Level 2' functions. This analysis revealed an over-representation of four categories and under-representation in seven others (Table 4).

Highly conserved genes that encode components of the ribosome and genes involved in peptidoglycan biosynthesis show little evidence of detectable recombination. Conversely, many genes involved in motility and chemotaxis undergo allelic substitution. Chemotaxis may also be related to elevated recombination detected among genes encoding components of phosphotransferase transport systems (PTSs) since these genes can double as sensors for substrates such as glucose and mannose [47].

Genes involved in basic processing of cellular information, such as replication, transcription and translation, reveal an unexpected dichotomy: genes dedicated to RNA and protein metabolism are refractory to recombination, but genes involved with DNA replication, repair and recombination appear prone to allelic substitution. Equally surprising is a bias favoring evident recombination among genes involved in small molecule biosynthesis. Examples of biosynthetic genes that support the pairings in topology ψ_*KC *_include members of the aromatic amino acid pathway (*aroP*, *aroD*, and *aroG*) as well as the pyrimidine producing *carB *(also known as *pyrA*). SND clusters supporting topology ψ_*KO *_are present in *pyrI*, *pyrB*, and several genes in the histidine operon. Finally, *purD*, *purF*, *leuDC*, *modABC*, and two genes in the *trp *operon (Figure 4) contain clusters compatible with the clonal topology, but at much higher intensity than elsewhere in the genome.

### Mosaic operons and genes

**Figure 5:**
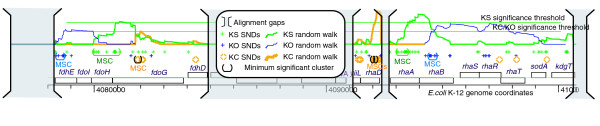
Mosaic operons and genes. Three of six *rha *genes (*rhaB*, *rhaA*, and *rhaD*) belong to an operon on the reverse strand. This operon is unusual because well-defined recombination events clearly fall within gene boundaries; *rhaD *contains two dense KC clusters, whereas *rhaA *and *rhaB *contain predominantly KS and KO SNDs, respectively. In a nearby operon consisting of *fdoG*, *fdoH*, *fdoI*, and *fdhE*, there has been a KC intragenic recombination event with *fdoG *a mosaic, resulting from two recombination events, one of which is shared with *fdoH*.

With over 216 recombined segments intersecting 271 genes, this group of *E. coli *genomes is truly a patchwork of its constituent members. Although genes within the *trp *and *his *operons contain multiple clusters of the same pattern (KS for *trp*, KO for *his*), such uniformity across operons is atypical [48]. Figure 5 shows a short stretch of aligned sequence containing two mosaic operons.

**Figure 6:**
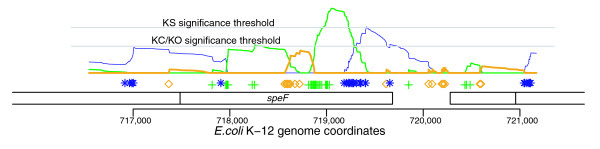
Random walk plots for positive local scores in the vicinity of the *speF *gene. *SpeF *is a mosaic gene by virtue of its KS and KO clusters. Note the small cluster of KC SNDs appears to divide a large KS segment near coordinate 718,600. This short KC spike, though not statistically significant on a whole genome scale, would undoubtedly pass a single gene substitution distribution type test.

Besides *fdoG *(shown in Figure 5), six other genes - *polB*, *mutS*, *speF*, *recG*, *actP*, and *yfaL *- show evidence of mosaicism. Three of these genes - *polB*, *mutS*, and *recG *- are informational genes involved in DNA replication and repair. Each mosaic gene contains two minimum significant clusters generated by different partition patterns. A closer inspection of one of these genes, *speF*, suggests that all three phylogenetic signals may be present, as shown in Figure 6.

Other mosaic genes undoubtedly exist within these strains, but their phylogenetic signal is too short or too weak to register in a genome-wide scan. Full genome scans come at a cost; one must sacrifice sensitivity to maintain specificity. At present, we are content to underestimate the true amount of recombination in order to eliminate false positives.

## Discussion

Natural transformation, transduction, and conjugation are three mechanisms for transporting foreign DNA into the cell. The relative contribution of each mechanism varies from species to species. For example, transformation is the dominant mode of transfer in bacteria such as *Neisseria meningitidis *and *Helicobacter pylori *that are naturally competent, that is, able to absorb small pieces of naked DNA. As *E. coli *is competent only under extreme conditions, typically in the laboratory, it is expected that this form of transformation may play a minor role in nature. Exogenous DNA can also enter via phage transduction or conjugation, which are expected to be the primary source of exogenous DNA for *E. coli*. Transducing phages can deliver large fragments of genomic DNA from their previous bacterial host into a recipient strain. DNA transferred via conjugative mechanisms can be even larger.

The lengths of recombined segments reported in the previous section are typically short. Half the intervals are shorter than 1 kb, and 80% are less than 2 kb. DNA fragments delivered by transducing phages might be expected to be considerably larger (30 to 60 kb). The size differential between entrance and incorporation molecules has been partially reconciled by experiments in which site-specific DNA was packaged into phages and transduced into K-12 cells [49]. Screening for recombinants in the proximity of the *trp *operon, the authors found average replacement sizes to be in the 8 to 14 kb range. Moreover, multiple replacements were detected in some instances. In a follow-up paper [1], the level of sequence dissimilarity (from 1% to 3%) between recipient and donor strains was shown to correlate with the degree of abridgement by restriction endonucleases. The length of a typical recombinant in our study is still an order of magnitude less than that reported by McKane and Milkman [49], but they based their conclusions on restriction site analysis, which has a limited ability to detect short fragments. Actual incorporations in their experiments could conceivably have been more frequent and shorter. Overlapping recombination events at particular sites are also likely to contribute to the net reductions in observed incorporation sizes.

**Figure 7:**
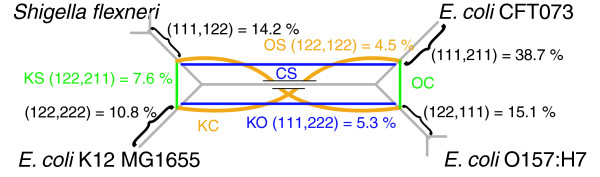
Percentage of SNDs supporting each of three topologies in a phylogenetic network for six *E. coli *genomes (four OTUs). Black lines describe the 'species' topology. Green, blue, and orange lines indicate the alternative pairings of sister taxa that result from KS, KO, and KC recombinations, respectively. Also shown is the percentage of SNDs supporting each bipartition in Table 1.

Our approach detects significant clusters of phylogenetically informative SNDs, but does not tell us which lineages participated in the recombination. When presented with four OTUs, recombination is possible between six undirected donor-recipient pairs: KO, CS, KS, OC, KC, and OS. These alternative histories can be jointly represented as a phylogenetic network (Figure 7).

**Figure 8:**
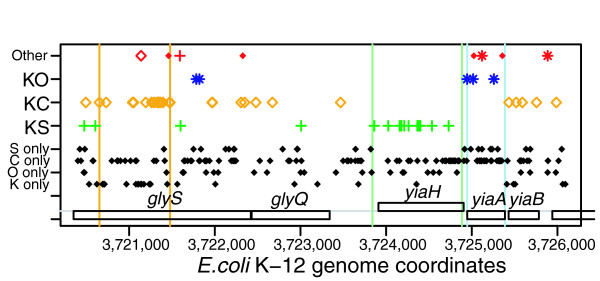
The location of all SNDs in a 5 kb region. In clusters demarcated by colored lines, note the corresponding absence of two more common types of SNDs. Three diamonds in lighter shades of blue, green, and red are compatible tri-partitions (see Additional data file 1). Colored lines demarcate regions where the absence of lineage-specific SNDs is offset by an increase in the corresponding recombinant pattern (for example, in *yiaA*, no K-12 or *S. flexneri *only SNDs).

For example, a high scoring KC segment indicates that the donor and recipient lineages are either K-12 and CFT, or O157:H7 and *S. flexneri*. Exactly which pair of lineages is involved in the transfer can sometimes be determined by examining the joint distribution of all seven SND patterns. Recombinant activity in *glyS *and the four genes to its right is illustrated in Figure 8.

The colored intervals in Figure 7 share a common feature: the presence of topologically informative SNDs is accompanied by the absence of SNDs from two paired sister taxa. For example, no 'O157 only' or '*Shigella *only' SNDs are present in the KC/OS interval inside *glyS*, strongly suggesting that the O157:H7 and *S. flexneri *lineages were involved in the transfer. The other two intervals coincide with gene boundaries. When viewed in isolation, the genes *yiaA *and *yiaH *appear to be reasonable candidates for recombination. Yet only the KC recombinant inside the *glyS *gene is detectable by our whole genome significance thresholds.

Sequence divergence can reduce the likelihood that homologous recombination occurs between orthologous genes, but does not address the underlying mechanisms that lead to divergence in the presence of rampant recombination. The restriction of different lineages of bacteria to distinct niches could act to prevent gene flow, but in the case of *E. coli *and *Salmonella*, the niches overlap. The barriers to exchange might also reflect more active exclusion of foreign DNA by mechanisms such as restriction enzyme expression. Perhaps the most appealing explanation for the phenomenon would invoke the activity of bacteriophages, transposons and conjugation-promoting elements as the key determinants of recombinational potential between taxa. Given the propensity of these mobile elements to participate in genetic exchange within species and their often narrow host ranges, we might expect that they promote recombination within a species but cannot transfer to more diverse organisms. The lack of extensive recombination of orthologous sequences between species may result from a competition between bacteria and phage that can activate rapid evolution of barriers to phage infection. Our estimate for a higher rate of homologous recombination among *E. coli *underscores the discrepancy between rates of intraspecies recombination, which appear to be quite common, and rates of recombination of orthologous genes between species such as *E. coli *and *Salmonella*, which appear to be much less frequent [2].

Earlier comparisons of different *E. coli *strains [1,11,14,50] found recombination among several distinct sets of genes. The affected genes in these studies were not randomly selected and may not have been representative of the shared gene complement. Although our method surveys all genes, the genomes we compared are heavily skewed towards human pathogens. As additional *E. coli *strains are sequenced, the role of homologous recombination in bacterial genome evolution will become clearer, and may force reassessment of traditional methods for describing relationships among bacterial taxa [8,51].

Our analytical methods are straightforward here because the number of unrooted topologies is the same as the number of topologically informative bipartitions. This correspondence decays exponentially as more operational taxonomic units are added. Sometimes going from four OTUs to five requires a new analytic procedure (for example, see [52]). We leave the challenging problem of extension to more taxa for future work.

## Conclusion

We demonstrate that the rate of intraspecies recombination in *E. coli *is much higher than previously appreciated and may show a bias for certain types of genes. The described method provides high-specificity, conservative inference of past recombination events.

## Materials and methods

The Mauve alignment tool produces an output file containing separate alignments for each locally collinear block. Concatenation of LCBs results in a G × M matrix of nucleotides and gap symbols, where G is the number of genomes and M is the length of gapped alignments across all blocks. Each matrix column represents one site in the consolidated alignment. Restricting attention to columns containing at least one nucleotide difference but no gaps results in a G × M' sub-matrix Δ composed solely of single nucleotide differences. Automated screening of the Mauve alignment (Figure 1) filtered out SNDs in regions of poor alignment quality, resulting in a Δ with dimension 6 by 130,008 (see Appendix 4 in Additional data file 1 for protocol employed).

Numerous scoring schemes have been devised to identify and assess the statistical significance of molecular sequence features on a genomic scale [53,54]. One general approach calculates average scores within a sliding window (for example, [55,56]). We use an equally versatile method that computes cumulative scores based on a score function, evaluated at each column of Δ (see [39] for other applications).

Let Ξ = {*KS*, *KC*, *KO*} represent the three discordant SND patterns in Table 1, and let ψ_ξ _be the unrooted topology compatible with pattern ξ ∈ Ξ. We define three complementary score functions on SNDs to filter conflicting phylogenetic signals:



where *s *is a SND and φ(*s*) is the corresponding partition pattern in Table 1, and *D *= 13. For a given ξ ∈ Ξ, the cumulative score at the *nth *column in Δ is the partial sum:



These score functions share a key characteristic of alignment scoring schemes; both generate high scoring segments that identify regions of interest. In the case of alignments, a high score segment represents a likely sequence homology. A significant difference between our analysis and sequence alignment is that substitution matrices are empirically derived from a test set (for example, PAM or BLOSUM). Here, *D *is not a parameter in an underlying stochastic model of evolution, but rather a tuning parameter in a diagnostic specifically designed to detect recombination. The value *D *= 13 was inspired by the observation that the most frequent topologically informative pattern, KS, has an observed frequency of 7.6%, approximately the reciprocal of 13. Alternative integer values were tried and rejected.

Score functions generate high scoring segments whenever they encounter a cluster of SND patterns supporting one topology but are discordant with other choices. For a given topology ψ_ξ_, we define *Score*_ξ_(η) to take on positive values when pattern η is ξ and negative values otherwise (η ≠ ξ,). As discordant patterns are antithetical to one another, their weights should be equal to but opposite from the one being scanned. Neutral SND patterns are not individually disruptive to the underlying signal, but in aggregate they degrade the signal. These non-informative patterns are down-weighted and made integer-valued as in substitution matrices.

Hence, a large local score - the equivalent of a high scoring segment - is evidence for recombination between two of the lineages paired by ξ (for example, ξ = *KS *associates K-12 with *S. flexneri *and O157:H7 with CFT).

Random walk plots connect the dots' between partial sums that are computed from SNDs as they occur in Δ. By contrast, random walks are translation invariant stochastic processes governed by the relative frequencies in Δ, irrespective of order. We augment the random walk transition probabilities with an additional 'terminator' state. Terminators break a global alignment into several smaller sub-alignments, and are used to represent alignment fragmentation caused by 'large' gaps (>15 bp in one lineage), spurious alignments, or LCB boundaries (Figure 1). Accordingly, for each ξ ∈ Ξ, random walk increments are distributed according to the following probabilities:



where *D *= 13, π_*KO *_= 0.048, π_*KS *_= 0.076, π_*OS *_= 0.045, π_*other *_= 0.826, π_*break *_= 0.005 and 

**Figure 9:**
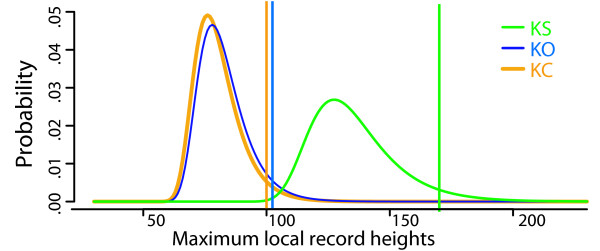
Statistical justification of threshold values - 100, 100, and 170 for topologies KO, KC, and KS, respectively - used to identify recombination events. Values on the x-axis are maximal local scores. EVD probability densities for the maximum maximal local score attained by random walks of length M' appear as bell-shaped curves with a pronounced skew to the right. Threshold values, demarcated by vertical lines, correspond to conservative significance levels (α = 0.05) for these distributions.

Since the expected value *E*(*X*^ξ^) < 0,∀ξ, sums of these identically distributed variables generate transient random walks. Random stopping times, defined recursively by:



form a strictly decreasing set of ladder points. Though *S*_*k *_depends on ξ, we suppress it for ease of exposition. The horizontal distances between consecutive ladder points: τ_*k*+1 _- τ_*k*_, are called ladder epochs. The local record height (LRH) of the *kth *epoch is defined by:



Ladder epochs measure the size of a high scoring segment in SND units rather than base pairs (chain length M' versus M). The number of ladder epochs in a random walk of size *N *is denoted by Λ(*N*). The distribution of the maximum value in a sequence of local record heights is an extreme value distribution (EVD) with parameterization:



Here μ is the positive solution of an equation involving the moment generating function:



The value of μ is solved for numerically. For ψ_*KC*_, the equation:

*mgf*_*KC*_(μ) = 0.045*e*^13μ ^+ .124*e*^-13μ ^+ .826*e*^-μ ^+ .005*e*^-100,000μ ^= 1

has a positive solution at μ = 0.1354 (μ = 0 is a trivial solution). The value of *K *can be computed as a rapidly converging infinite sum (see appendix of [39]). We chose instead to simulate 2,000 random walks of size *N *= 10,000 using the statistical package R [57]. The largest local record height attained over the course of each simulation is saved. The functional form of the EVD (equation 1) is then fit to a probability histogram of 2,000 stored maxima. The estimated values of K and Λ are combined with an *N *= M' to adjust for the actual alignment size (M' = 129,000 after excluding the atypical region) in each EVD. The densities of the three EVDs are plotted in Figure 9.

Ladder points, ladder epochs, and local record heights are easily computed with a few simple R commands. Finding minimal significant clusters - a smallest possible cluster of SNDs with a significant score - is more challenging. A naïve approach takes each SND within a high scoring segment as the start of some local score, then iteratively adds successive terms to local scores in parallel until one of the sums exceeds the threshold. The SNDs producing that sum constitute the first MSC. The process continues on the remaining sums to seek out additional, non-overlapping MSCs. The algorithm is *O*(*n*^2^) in the number of SNDs. Such a brute force approach works here because alignment gaps split the problem into 186 small pieces, the largest of which contains fewer than 700 SNDs.

### Accession numbers

Deposited accession numbers are: *Escherichia coli *CFT073 [GenBank:AE014075]; *Escherichia coli *K-12 MG1655 [GenBank:U00096]; *Escherichia coli *O157:H7: RIMD0509952 (Sakai) [GenBank:BA000007]; *Escherichia coli *O157:H7: EDL933: [GenBank:AE005174]; *Shigella flexneri *2a str.2457T: [GenBank:AE014073]; *Shigella flexneri *2a str.301: [GenBank:AE005674].

## Additional data files

The following additional data are available with the online version of this paper. Additional data file 1 is a PDF document containing five appendices. Appendix 1 shows the distribution of rare SNDS supplementing Table 1. Appendix 2 shows the comparative analysis of the large atypical region. Appendix 3 shows genes uniquely present in 13 γ-proteobacteria that have undergone homologous recombination between the four lineages of *E. coli*. Appendix 4 contains the screening protocols used to delete erroneous alignment of non-homologous sequence. Appendix 5 shows the local deviation in the rate of mutation among the six genomes. Additional data file 2 is a spreadsheet enumerating all HSS, MSC, and affected genes in this analysis. Additional data file 3 is a text file of all 130,008 SNDs by pattern and location in K-12 MG1655 coordinates.

## Supplementary Material

Additional File 1 Five appendices. Click here for file

Additional File 2 Enumeration of all HSS, MSC, and affected genes in this analysis. Click here for file

Additional File 3 All 130,008 SNDs by pattern and location in K-12 MG1655 coordinates. Click here for file

## Figures and Tables

**Table 1:** Frequency of common patterns of single nucleotide differences

Bipartition (Split)	Pattern KOOCSS	Number of SNDs	Relative frequency
((KSSOO) C)	111211	50,354	38.73
((KSSC)(OO))	122111	19,678	15.14
((KOOC)(SS))	111122	18,490	14.22
(K(OOSSC))	122222	14,115	10.86
((KSS)(OOC)) = KS	122211	9,882	7.60
((KOO)(SSC)) = KO	111222	6,890	5.30
((KC)(OOSS)) = KC	122122	5,874	4.52

Common single nucleotide differences have two alleles. Each such nucleotide difference separates the six genomes into two classes. Pattern codes are represented as 6-tuples of ones and twos (for allele 1 and allele 2) in the following order: (K) *E. coli *K-12 MG1655, (O) *E. coli *O157:H7 EDL933, (O) *E. coli *O157:H7 Sakai strain RIMD0509952, (C) *E. coli *CFT073, (S) *Shigella flexneri *2A 301, and (S) *Shigella flexneri *2A 2457T. By convention, K-12 is always allele one. For brevity, key groupings are denoted as KS, KO, or KC. The remaining 3.6% SNDs come in over 50 different patterns, including one quadripartition. See appendix 1 in Additional data file 1 for additional frequencies.

**Table 2:** Distribution of scores of significant segments for discordant bipartitions

Bipartition pattern	Number of segments exceeding a given HSS threshold of 100				
	
	101-110	111-125	126-200	>200	Total
KO (CS)	13	17	25	7	64
KC (OS)	14	16	18	5	53

**Table 3:** Distribution of scores for KS (OC) high scoring segments

Pattern	Number of segments exceeding threshold of 170					
	
	170-200	201-220	221-250	251-400	>400	Total
KS (OC)	15	13	15	18	7	68

**Table 4:** MulitFun categories exhibiting unusual levels of allelic substitution among the four major lineages

HR detected	Genes	Percent recombined	χ^2 ^score	Multi-Fun Level 2 categories
5	144	3.5	4.52	Ribosome and peptidoglycan structure
10	237	4.2	5.47	Cell division, cell protection, and adaptation to stress
14	279	5.0	4.35	Protein-related information
20	329	6.1	2.94	RNA-related information
386	4,035	9.6	Not reported	All other functions (including unknown)
48	357	13.5	9.24	Building block biosynthesis
16	109	13.8	3.21	DNA-related information
7	40	17.5	3.56	Group translocators (PTS)
9	46	19.6	6.24	Motility

Categories with few members such as ribosome and peptidoglycan structure are combined together, as are three types of cell processes. We computed a χ^2 ^goodness-of-fit statistic for each category, but do not report *p *values because dependencies exist between categories.

## References

[B1] Milkman R (1997). Recombination and population structure in *Escherichia coli*.. Genetics.

[B2] Daubin V, Moran NA, Ochman H (2003). Phylogenetics and the cohesion of bacterial genomes.. Science.

[B3] Feil EJ, Maiden MC, Achtman M, Spratt BG (1999). The relative contributions of recombination and mutation to the divergence of clones of *Neisseria meningitidis*.. Mol Biol Evol.

[B4] Spratt BG, Hanage WP, Feil EJ (2001). The relative contributions of recombination and point mutation to the diversification of bacterial clones.. Curr Opin Microbiol.

[B5] Gogarten JP, Doolittle WF, Lawrence JG (2002). Prokaryotic evolution in light of gene transfer.. Mol Biol Evol.

[B6] Lawrence JG, Hendrickson H (2003). Lateral gene transfer: when will adolescence end?. Mol Microbiol.

[B7] Lerat E, Daubin V, Moran NA (2003). From gene trees to organismal phylogeny in prokaryotes: the case of the gamma-proteobacteria.. PLoS Biol.

[B8] Ochman H, Lerat E, Daubin V (2005). Examining bacterial species under the specter of gene transfer and exchange.. Proc Natl Acad Sci USA.

[B9] Ge F, Wang L-S, Kim J (2005). The cobweb of life revealed by genome-scale estimates of horizontal gene transfer.. PLoS Biol.

[B10] Beiko RG, Harlow TJ, Ragan MA (2005). Highways of gene sharing in prokaryotes.. Proc Natl Acad Sci USA.

[B11] Dykhuizen DE, Green L (1991). Recombination in *Escherichia coli* and the definition of biological species.. J Bacteriol.

[B12] Bowler LD, Zhang QY, Riou JY, Spratt BG (1994). Interspecies recombination between the penA genes of *Neisseria meningitidis* and commensal *Neisseria* species during the emergence of penicillin resistance in *N. meningitidis*: natural events and laboratory simulation.. J Bacteriol.

[B13] Suerbaum S, Smith JM, Bapumia K, Morelli G, Smith NH, Kunstmann E, Dyrek I, Achtman M (1998). Free recombination within *Helicobacter pylori*.. Proc Natl Acad Sci USA.

[B14] Reid SD, Herbelin CJ, Bumbaugh AC, Selander RK, Whittam TS (2000). Parallel evolution of virulence in pathogenic *Escherichia coli*.. Nature.

[B15] Carver TJ, Rutherford KM, Berriman M, Rajandream MA, Barrell BG, Parkhill J (2005). ACT: the Artemis Comparison Tool.. Bioinformatics.

[B16] Kurtz S, Phillippy A, Delcher AL, Smoot M, Shumway M, Antonescu C, Salzberg SL (2004). Versatile and open software for comparing large genomes.. Genome Biol.

[B17] Darling ACE, Mau B, Blattner FR, Perna NT (2004). Mauve: multiple alignment of conserved genomic sequence with rearrangements.. Genome Res.

[B18] Perna NT, Plunkett G, Burland V, Mau B, Glasner JD, Rose DJ, Mayhew GF, Evans PS, Gregor J, Kirkpatrick HA (2001). Genome sequence of enterohaemorrhagic *Escherichia coli* O157:H7.. Nature.

[B19] Parkhill J, Dougan G, James KD, Thomson NR, Pickard D, Wain J, Churcher C, Mungall KL, Bentley SD, Holden MT (2001). Complete genome sequence of a multiple drug resistant *Salmonella enterica* serovar Typhi CT18.. Nature.

[B20] Tettelin H, Masignani V, Cieslewicz MJ, Donati C, Medini D, Ward NL, Angiuoli SV, Crabtree J, Jones AL, Durkin AS (2005). Genome analysis of multiple pathogenic isolates of *Streptococcus agalactiae*: Implications for the microbial "pan-genome".. Proc Natl Acad Sci USA.

[B21] Hsiao WW, Ung K, Aeschliman D, Bryan J, Finlay BB, Brinkman FS (2005). Evidence of a Large Novel Gene Pool Associated with Prokaryotic Genomic Islands.. PLoS Genet.

[B22] Posada D, Crandall KA, Holmes EC (2002). Recombination in evolutionary genomics.. Annu Rev Genet.

[B23] Graham J, McNeney B, Seillier-Moiseiwitsch F (2005). Stepwise detection of recombination breakpoints in sequence alignments.. Bioinformatics.

[B24] Stephens JC (1985). Statistical methods of DNA sequence analysis: detection of intragenic recombination or gene conversion.. Mol Biol Evol.

[B25] Maynard Smith J, Smith NH (1998). Detecting recombination from gene trees.. Mol Biol Evol.

[B26] Qiu WG, Schutzer SE, Bruno JF, Attie O, Xu Y, Dunn JJ, Fraser CM, Casjens SR, Luft BJ (2004). Genetic exchange and plasmid transfers in *Borrelia burgdorferi sensu stricto* revealed by three-way genome comparisons and multilocus sequence typing.. Proc Natl Acad Sci USA.

[B27] Sawyer S (1989). Statistical tests for detecting gene conversion.. Mol Biol Evol.

[B28] Worobey M (2001). A novel approach to detecting and measuring recombination: new insights into evolution in viruses, bacteria, and mitochondria.. Mol Biol Evol.

[B29] Grassly NC, Holmes EC (1997). A likelihood method for the detection of selection and recombination using nucleotide sequences.. Mol Biol Evol.

[B30] Husmeier D, McGuire G (2002). Detecting recombination with MCMC.. Bioinformatics.

[B31] McGuire G, Wright F (2000). TOPAL 2.0: improved detection of mosaic sequences within multiple alignments.. Bioinformatics.

[B32] Minin VN, Dorman KS, Fang F, Suchard MA (2005). Dual multiple change-point model leads to more accurate recombination detection.. Bioinformatics.

[B33] Posada D, Crandall KA (2001). Evaluation of methods for detecting recombination from DNA sequences: computer simulations.. Proc Natl Acad Sci USA.

[B34] Raymond J, Zhaxybayeva O, Gogarten JP, Gerdes SY, Blankenship RE (2002). Whole-genome analysis of photosynthetic prokaryotes.. Science.

[B35] Harlow TJ, Gogarten JP, Ragan MA (2004). A hybrid clustering approach to recognition of protein families in 114 microbial genomes.. BMC Bioinformatics.

[B36] Mau B, Newton MA, Larget B (1999). Bayesian phylogenetic inference via Markov chain Monte Carlo methods.. Biometrics.

[B37] Ronquist F, Huelsenbeck JP (2003). MrBayes 3: Bayesian phylogenetic inference under mixed models.. Bioinformatics.

[B38] Altschul SF, Gish W, Miller W, Myers EW, Lipman DJ (1990). Basic local alignment search tool.. J Mol Biol.

[B39] Karlin S, Altschul SF (1990). Methods for assessing the statistical significance of molecular sequence features by using general scoring schemes.. Proc Natl Acad Sci USA.

[B40] Blattner FR, Plunkett G, Bloch CA, Perna NT, Burland V, Riley M, Collado-Vides J, Glasner JD, Rode CK, Mayhew GF (1997). The complete genome sequence of *Escherichia coli* K-12.. Science.

[B41] Wei J, Goldberg MB, Burland V, Venkatesan MM, Deng W, Fournier G, Mayhew GF, Plunkett G, Rose DJ, Darling A (2003). Complete genome sequence and comparative genomics of *Shigella flexneri* serotype 2a strain 2457T.. Infect Immun.

[B42] Jin Q, Yuan Z, Xu J, Wang Y, Shen Y, Lu W, Wang J, Liu H, Yang J, Yang F (2002). Genome sequence of *Shigella flexneri* 2a: insights into pathogenicity through comparison with genomes of *Escherichia coli* K12 and O157.. Nucleic Acids Res.

[B43] Hayashi T, Makino K, Ohnishi M, Kurokawa K, Ishii K, Yokoyama K, Han CG, Ohtsubo E, Nakayama K, Murata T (2001). Complete genome sequence of enterohemorrhagic *Escherichia coli* O157:H7 and genomic comparison with a laboratory strain K-12.. DNA Res.

[B44] Welch RA, Burland V, Plunkett G, Redford P, Roesch P, Rasko D, Buckles EL, Liou SR, Boutin A, Hackett J (2002). Extensive mosaic structure revealed by the complete genome sequence of uropathogenic *Escherichia coli*.. Proc Natl Acad Sci USA.

[B45] Stoltzfus A, Leslie JF, Milkman R (1988). Molecular evolution of the *Escherichia coli* chromosome. I. Analysis of structure and natural variation in a previously uncharacterized region between trp and tonB.. Genetics.

[B46] Serres MH, Riley M (2000). MultiFun, a multifunctional classification scheme for *Escherichia coli* K-12 gene products.. Microb Comp Genomics.

[B47] Zeppenfeld T, Larisch C, Lengeler JW, Jahreis K (2000). Glucose transporter mutants of *Escherichia coli* K-12 with changes in substrate recognition of IICB(Glc) and induction behavior of the ptsG gene.. J Bacteriol.

[B48] Omelchenko MV, Makarova KS, Wolf YI, Rogozin IB, Koonin EV (2003). Evolution of mosaic operons by horizontal gene transfer and gene displacement *in situ*.. Genome Biol.

[B49] McKane M, Milkman R (1995). Transduction, restriction and recombination patterns in *Escherichia coli*.. Genetics.

[B50] Guttman DS, Dykhuizen DE (1994). Clonal divergence in *Escherichia coli* as a result of recombination, not mutation.. Science.

[B51] Feil EJ, Spratt BG (2001). Recombination and the population structures of bacterial pathogens.. Annu Rev Microbiol.

[B52] Zhaxybayeva O, Hamel L, Raymond J, Gogarten JP (2004). Visualization of the phylogenetic content of five genomes using dekapentagonal maps.. Genome Biol.

[B53] Karlin S, Brendel V (1992). Chance and statistical significance in protein and DNA sequence analysis.. Science.

[B54] Karlin S, Bucher P, Brendel V, Altschul SF (1991). Statistical methods and insights for protein and DNA sequences.. Annu Rev Biophys Biophys Chem.

[B55] Lobry JR (1996). Asymmetric substitution patterns in the two DNA strands of bacteria.. Mol Biol Evol.

[B56] Scherer S, McPeek MS, Speed TP (1994). Atypical regions in large genomic DNA sequences.. Proc Natl Acad Sci USA.

[B57] The R Project for Statistical Computing. http://www.r-project.org/.

