# Supplementary material for: Genome-wide detection and analysis of homologous recombination among sequenced strains of Escherichia coli
Source: Genome Biol. 2006 May 31;7(5):R44. doi: 10.1186/gb-2006-7-5-r44 (PMC1779527; doi:10.1186/gb-2006-7-5-r44)
Supplement: Additional File 1 — Five appendices. [file gb-2006-7-5-r44-S1.pdf]

Appendix 1: The distribution of rare SNDs

Partition Structure	Pattern Code	Number of SNDs
KSS / OO / C ~ KS	122311	670
KOO / SS / C ~ KO	111233	473
KC / OO / SS ~ KC	122133	208
K / OOC / SS ~ OC	122233	184
K / OO / CSS ~ CS	122333	236
K / OOSS / C ~ OS	122322	459
K / OO / SS / C	122344	19
O157:H7 EDL only	121111	1268
O157:H7 Sakai only	112111	73
S. flexneri 2A 301 only	111121	722
S. flexneri 2A 2457 only	111112	210
Other miscellaneous	******	295

Table A1: Rare partition patterns, comprising 3.6 % of all SNDs, that are not displayed in Table 1. The elevated frequency of EDL strain is due to over 1200 ambiguities its genome sequence. Note that colored tri-partitions that isolate CFT (KS, KO, and OS) occur twice as often as those that divide complementary pairs (OC, CS, KC)

Appendix 2: Analysis of large atypical region.

One recombination event in which the participating strains and the direction of transfer can be confidently assigned involves the large atypical region. Relative SND frequencies and their deviations from the overall frequencies in Table 1 are arrayed below, along with the flanking regions alluded to earlier.

	459 SNDs 12.1 kb	Core Region 1,364 SNDs / 37.6 kb		809 SNDs 15.9 kb
SND Pattern	Relative frequency	Relative frequency	Change from norm	Relative frequency
CFT only	22.2 %	7.8 %	-31.5 %	13.3 %
KO (or CS)	16.9 %	37.1 %	+32.2 %	16.1 %
K-12 only	14.8 %	18.8 %	+8.0 %	19.6 %
KS (or OC)	2.5 %	0.5 %	-7.2 %	4.1 %
O157 only	17.3 %	18.5 %	+3.4 %	20.9 %
OS (or KC)	3.2 %	0.8 %	-3.8 %	3.5 %

Table A2: Changes in pattern composition between 1,994.2 and 2,099.4 kb in K-12 MG1655 coordinates. Left flanking region extends from *sdiA* to *yedF* and contains 7 *fli* genes. The core region (*fliE* to *cobU*) encompasses the remaining 14 *fli* genes. Deviations are computed from genome-wide averages outside these regions. The right flanking region, situated between *sbcB* and *gnd,* contains the *his* operon.

The central region in Table 4 exhibits a striking shift in SND frequencies. A huge decrease in SNDs unique to CFT is offset by a matching increase in the number of KO SNDs. Significant shifts also occur between “K-12 only” and KS patterns (+8.0% vs. –7.2%), and “O157 only” and KC patterns (+3.4% vs. –3.8%).

Homologous recombination of a 40 kb segment of CFT sequence into an ancestral *Shigella flexneri* genome explains the observed frequency differences between the core region and the clonal frame, since accumulated ancestral “CFT only” SNDs are shared with *Shigella flexneri.* Had the exchange gone in the other direction, evidence of these relatively ancient SNDs would have been expunged from the historical record.

Appendix 3: Genes uniquely present in 13 γ -Proteobacteria that have undergone homologous recombination between the four lineages of *E. coli*

	alas	*apaH*	*nth*	*gltX*	*glyQ*	*glyS*	*ksgA*	*mviN*	Total
K only	12	7	3	0	2	18	4	1	47
O only	2	4	3	8	4	9	1	6	37
C only	12	11	6	15	6	33	7	2	92
S only	8	2	3	2	3	9	3	7	37
KS	0	0	0	**11**	1	3	0	**9**	24
KC	**9**	**4**	0	0	**2**	**21**	**3**	0	39
KO	2	0	**4**	1	0	2	1	1	11
misc	2	0	1	4	0	5	0	2	14
Total SNDs	47	28	20	41	18	100	19	28	301

Table A3: Distribution of SND patterns in genes that are identified in Supporting Information of [7] as well as our own study. Dominant discordant patterns are denoted in bold font. NB: *mviN* was one of two (out of 205) unique orthologs that generated a discordant gene tree in [7].

Hence, in contrast to what has been observed across species comparisons of γ-proteobacteria genomes, housekeeping genes within a particular species can experience homologous recombination.

The highly conserved gene *rpmH,* which encodes the small ribosomal protein known as L34, was classified (by our method) as having undergone recombination. The DNA sequence of *rpmH* is in fact identical in all six strains, but is bordered of each side by genes that appear to share a single recombination event that spans *rpmH.*

Appendix 4: Screening protocols used to delete erroneous alignment of non-homologous sequence

The uncurated Mauve alignment of the six genomes generated 174,494 columns with at least one mismatch, and 7035 gaps consisting of over 4 million gap characters. Gaps varied in size from an 87.5 kb insertion in both O157:H7 lineages to 805 singletons distributed throughout the alignment. Alignment subroutines called by Mauve (ClustalW and Muscle) have a tendency to align small segments of non-homologous sequence situated between unambiguous alignment anchors. Consequently, a post-processing step was invoked to filter out false SNDs due to misalignments.

One simple filtration method consolidates small gaps by merging them into larger gaps whenever the intervening aligned region is below a fixed threshold size. Subsequent visual inspection revealed numerous instances where non-homologous sequences of similar lengths remained erroneously aligned. Screening by gap consolidation alone is too conservative; a more aggressive filtration method is necessary. The automated screening process we settled on first identifies long runs of suspect alignment consisting of clusters of mismatches and gaps separated by no more than five consecutive matches. The corresponding intervals, together with intervening segments less than 100 bps, were removed from the raw alignment, resulting in the 130,008 SNDs and 733 condensed gaps reported in the paper. In the process, valid sections of the alignment were invariably jettisoned to insure all residual differences were *bona fide* SNDs - the product of vertical or horizontal inheritance – and not an artifact of an erroneous alignment. Hence, specificity was maximized at the expense of sensitivity.

Appendix 5: Local deviation in the rate of mutation

In the data set presented, extreme local rate variability has been filtered out such that our predictions of recombined segments should be not compromised. There are two types of local rate variability that can compromise the analysis: (1) lineage specific and (2) locus specific (position in the genome).

1. Mutation rates are nearly consistent across lineages

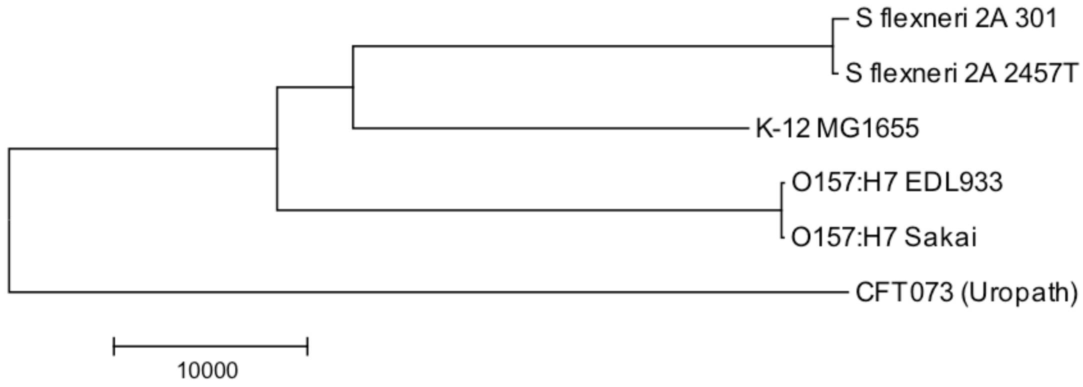

Figure A1: A whole genome phylogeny based on pairwise sequence differences in the 127,376 SNDs outside the 100 kb atypical region. The tree is constructed with a Neighbor-Joining algorithm as implemented in Mega 3.1.

Under the molecular clock hypothesis, one would expect all taxa to be contemporaneous. The phylogeny depicted in Figure A1 suggests that the genomes in our data set show only modest deviation from a constant nucleotide substitution rate among lineages.

A contributing factor for the elevated number of mutations present in the *Shigella flexneri* lineage is due to its high complement of pseudogenes relative to the other strains in this comparison. Pseudogenes no longer code for proteins, hence each base in the homologous sequence is subject to the same neutral selection usually reserved for nucleotides in the third codon position of functional genes. Table A4 reports the distribution of distances between consecutive lineage specific SNDs, modulo 3, as explained below.

k	K-12	Sakai	CFT	*Shigella flexneri* 2A Str. 301 vs.	(Str. 2457T)
0	60.46	60.87	66.63	52.61	(53.39)
1	20.16	19.60	17.21	24.27	(23.74)
2	19.38	19.52	16.16	23.12	(22.87)
Total	8,318	11,584	24,937	13,543	(12,991)

Table A4: The first row denotes the percentage of lineage specific SNDs that have inter-SND distances which are multiples of three. As an example, if s_1 and s_2 are two consecutive "K-12 only" SNDs (*i.e.* pattern 122222), then dist(s_1, s_2) = 3*m + k, where k=0,1, or 2, and m is a positive integer. The value of m is irrelevant; the value of k determines to which class each inter-SND distance is assigned. For k=0, both *Shigella flexneri* genomes have significantly lower relative frequencies than the other strains. On the other hand, the corresponding percentage for CFT is somewhat elevated (66.6%).

Since bacterial genome sequences are 85% coding, we argue that the row denoted by k=0 is a proxy for mutations occurring in the third codon position. The subsequent increase in relative frequencies in rows k=1 and k=2 for *Shigella flexneri* are hypothesized to occur within pseudogenes. Hence, the rate of spontaneous mutation may be the same for in *Shigella flexneri* as in K-12 and O157:H7 strains, but the selective pressures for repairing them are relaxed inside pseudogenes.

2. Regions of significant hypervariability are filtered out

Whole genome summaries obscure local (site-specific) variability by averaging it out. For example, genes coding for antigens undergo more rapid rates of mutation in pathogens in order to avoid host responses. By contrast, ribosomal RNAs are highly conserved across all domains of life.

In Appendix 4, the post-processing procedure used to screen the Mauve output for misalignment of non-homologous sequence is described. We now show that this step also filters out regions of significant hypervariability. Thus, our results are free of potential identification errors arising from locally elevated rates of mutation.

The following diagnostic, based on the same random walk principles developed in the main text, illustrates the effectiveness of our screening process in eliminating high-density SND regions as well as misalignments. We compare the density between two sets of SNDs: one generated by simple gap consolidation, resulting in 144,490 SNDs and 1,053 gaps, and aggressive filtering we actually employed. We construct random walk plots based on the distances between consecutive SNDs in K-12 coordinates: if the distance between SND s_i and s_{i+1} is d_i, the increment at the ith step is $x_i = 8 - d_i$. The random walk plot *per se* is not of interest here, but rather the local record heights (as with earlier our choice of D (=13), the value 8 in the increment was chosen by trial and error).

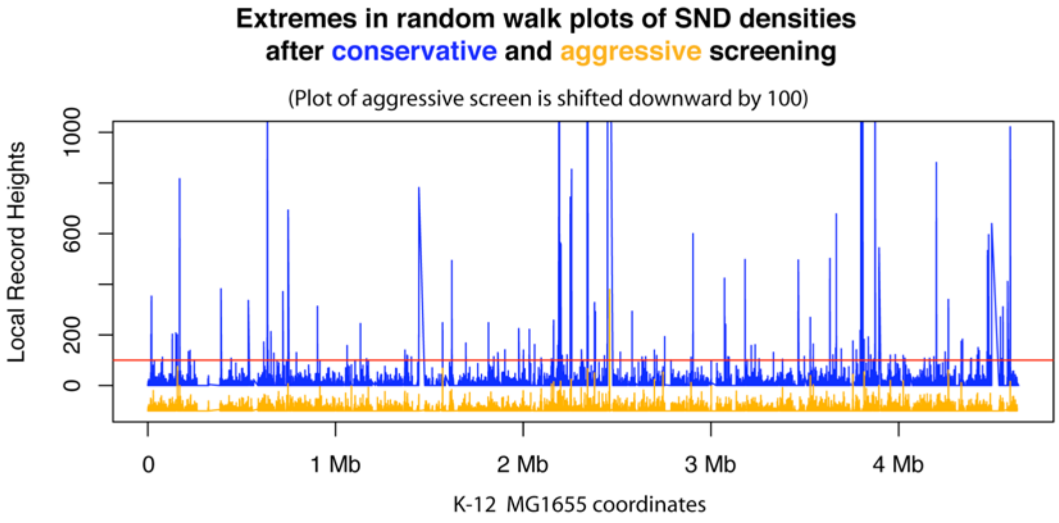

Figure A2: Comparison of local record heights based on random walk plots for two SND sets (of size 144,490 and 130,008). Large LRHs represent regions of high SND density. The scales are identical, but the aggressively screened SND set (130,008) is shifted down 100 bps for interpretative ease. The benchmark red horizontal line is set at +100 bps. Only once does the orange plot cross the red line (in the *fadL* gene at 2.45 Mb).

Hence, our protocol for eliminating potential misalignments has also removed dense SND intervals from consideration.
