# Supplementary material for: Genome-wide detection and analysis of homologous recombination among sequenced strains of Escherichia coli
Source: Genome Biol. 2006 May 31;7(5):R44. doi: 10.1186/gb-2006-7-5-r44 (PMC1779527; doi:10.1186/gb-2006-7-5-r44)
Supplement: Additional File 2 — Enumeration of all HSS, MSC, and affected genes in this analysis. [file gb-2006-7-5-r44-S2.pdf]

	Minimal Significant	Cluster								High Scoring Segments			
K-12 start	K-12 end	Length	Gene	bnum	Gene Length	MSC span	Inter-section	Lerat List		SNPs	Score	K-12 start	K-12 end
27810	27901	92	rihC	b0030	914	91	Insd		KO	53	321	27741	
27909	28059	151											28059
32183	32657	475	carB	b0033	3221	474	Insd		OS	109	301	32183	
32735	33077	343											33962
50468	53208	2741	apaH	b0049	842	2740	OvrR	***	OS	148	102	50468	
			apaG	b0050	377	2740	Span						
			ksgA	b0051	821	2740	Span	***					
			pdxA	b0052	989	2740	OvrL						53208
64277	64400	124	polB	b0060	2351	123	Insd		KO	37	124	64238	64400
64520	65380	861			2351	860	Insd		KS	86	190	64520	65726
74646	75114	469	tbpA	b0068	983	468	Insd		KO	73	140	74562	75114
79012	79811	800	leuD	b0071	605	799	OvrR		KS	138	177	79012	
			leuC	b0072	1400	799	OvrL						80015
116889	117069	181	hofB	b0107	1385	180	Insd		OS	178	178	116508	
120438	120506	69	aroP	b0112	1373	68	Insd						120519
133848	134217	370	acnB	b0118	2597	369	OvrR		KO	130	120	133728	134359
159488	159596	109	yadB	b0144	926	108	Insd		KS	240	312	159484	160019
213038	213481	444	tilS	b0188	1298	443	Insd		KO	80	144	212996	213481
219270	219704	435	yaeB	b0195	707	434	OvrR		KS	253	203	216223	219704
			rcsF	b0196	404	434	OvrL						
250391	250823	433	mbhA	b0230	785	432	Insd		OS	59	102	250391	250823
406544	407113	570	aroM	b0390	677	569	OvrL		KS	104	256	404331	407235
413193	413348	156	sbcC	b0397	3146	155	Insd		OS	138	220	412826	413678
489640	489820	181	priC	b0467	527	180	Insd		KS	231	205	489411	489820
501490	501532	43	ybaL	b0478	1676	42	Insd		KO	110	274	501487	
502036	502267	232											503047
529754	530125	372	ybbB	b0503	1094	371	Insd		KO	146	170	529526	530800
595235	596186	952	cusC	b0572	1373	951	Insd		KO	58	118	595193	596186
608911	609029	119	entD	b0583	629	118	Insd		OS	117	160	608605	609068
612294	613703	1410	fes	b0585	1124	1409	OvrR		OS	114	113	612135	
			entF	b0586	3881	1409	OvrL						613703
631692	632049	358	ybdH	b0599	1088	357	Insd		OS	98	116	631607	632049

694604	694746	143	ubiF	b0662	1175	142	Insd		OS	50	151	694604	694746
711803	713886	2084	ybfF	b0686	764	2083	OvrR		KO	88	104	711803	
			seqA	b0687	545	2083	Span						
			pgm	b0688	1640	2083	OvrL						713886
718837	718993	157	speF	b0693	2198	156	Insd		KS	39	242	718837	719050
719245	719413								KO		173	719188	719413
723858	724168	311	kdpC	b0696	572	310	Insd		KO	116	103	723858	724168
770403	770507	105							KS	18	207	770403	770507
771519	773178	1660	cydA	b0733	1571	1659	OvrR		KS	105	273	770605	
			cydB	b0734	1139	1659	OvrL						774716
785301	785664	364	aroG	b0754	1052	363	Insd		OS	58	108	785301	785664
794811	796429	1619	modA	b0763	773	1618	OvrR		KS	120	193	794811	
			modB	b0764	689	1618	Span						
			modC	b0765	1058	1618	OvrL						797018
805913	806426	514	ybhC	b0772	1283	513	Insd		OS	25	120	805913	806426
815366	815588	223	ybhK	b0780	908	222	Insd		KS	492	458	814995	
815591	816985	1395	moaA	b0781	989	1394	OvrL						817495
842204	843188	985	ybiN	b0807	1007	984	OvrR		KO	140	104	842204	
			ybiO	b0808	2360	984	OvrL						843188
853771	854082	312	ybiR	b0818	1118	311	OvrR		KO	119	106	853771	
			ybiS	b0819	920	311	OvrL						854082
893275	893998	724	potF	b0854	1112	723	Insd		KS	139	252	893254	895067
916657	916714	58	ybjD	b0876	1658	57	Insd		OS	179	310	916273	
916717	916807	91											916807
943833	944273	441	dmsC	b0896	863	440	OvrR		OS	88	136	943833	
			ycaC	b0897	626	440	OvrL						945371
952384	952501	118	pflB	b0903	2282	117	Insd		OS	162	178	951358	
955036	955168	133	ycaO	b0905	1769	132	Insd						953356
1007967	1008214	248	ycbY	b0948	2108	247	Insd		KO	81	122	1007878	1008232
1018943	1019017	75	ompA	b0957	1040	74	Insd		KO	46	278	1018943	
1019063	1019144	82											1019680
1084155	1084256	102	phoH	b1020	1064	101	OvrL		KO	56	384	1084119	
1084265	1084316	52											1084651
1127990	1132308	4319	mviN	b1069	1535	4318	OvrR	****	KS	160	258	1127681	
			flgN	b1070	416	4318	Span						
			flgM	b1071	293	4318	Span						

			flgA	b1072	659	4318	Span						
			flgB	b1073	416	4318	Span						
			flgC	b1074	404	4318	Span						
			flgD	b1075	695	4318	Span						
			flgE	b1076	1208	4318	OvrL						1132308
1142351	1142390	40	rne	b1084	3185	39	Insd		KO	119	143	1141585	1142390
1153951	1154894	944	yceG	b1097	1022	943	OvrR		OS	122	110	1153951	
			tmk	b1098	641	943	OvrL						1155343
1280912	1281185	274	narG	b1224	3743	273	Insd		KS	141	223	1280876	1281323
1314158	1315093	936	trpA	b1260	806	935	OvrL		KS	416	443	1314158	
1319366	1319791	426	trpD	b1263	1595	425	OvL						
			trpE	b1264	1562	425	OvR						1320726
1365045	1365159	115	pspF	b1303	992	114	Insd		KO	60	101	1365045	1365159
1368754	1368793	40	ycjM	b1309	1706	39	Insd		OS	53	141	1368742	1368793
1689270	1689389	120	ydgA	b1614	1508	119	OvrR		KS	53	202	1688108	1689389
1697521	1699267	1747	malX	b1621	1592	1746	OvrR		KS	116	179	1697521	
			malY	b1622	1172	1746	OvrL						1699267
1706885	1709842	2958	rnfC	b1629	2222	2957	OvrR		KO	166	106	1706885	
			rnfD	b1630	1058	2957	Span						
			rnfG	b1631	620	2957	Span						
			rnfE	b1632	695	2957	Span						
			nth	b1633	635	2957	OvrL	***					1709842
1716454	1716525	72	ydhH	b1640	1109	71	OvrL		OS	143	150	1716440	1717164
1765563	1765617	55	ydiJ	b1687	3056	54	Insd		KO	57	152	1765509	1765617
1771703	1773005	1303	ydiN	b1691	1271	1302	OvrR		OS	82	125	1771461	
			ydiB	b1692	866	1302	Span						
			aroD	b1693	758	1302	OvrL						1773005
1821705	1821798	94	ydjQ	b1741	887	93	Insd		OS	158	152	1821705	1822010
1830817	1831014	198	xthA	b1749	806	197	Insd		KS	170	280	1829519	1831014
1838980	1839136	157	ynjF	b1758	626	156	Insd		KS	66	251	1838980	1839301
1850992	1851360	369	ydjE	b1769	1358	368	Insd		KO	76	169	1850306	1851360
1889486	1889942	457							KS	122	172	1889486	1889942
1944717	1945449	733	yebB	b1862	701	732	OvrR		KO				
			ruvC	b1863	521	732	Span						
			yebC	b1864	740	732	OvrL						
1951402	1951659	258	yecO	b1870	743	257	OvrR		KO				

			yecP	b1871	971	257	OvrL					
1981435	1983659	2225	araH	b4460	989	2224	OvrR	KS	280	206	1979715	
			araG	b1900	1514	2224	Span					
			araF	b1901	989	2224	OvrL					1984062
****	The Atypical Region											
		478	sdiA	b1916	722	477						
		258			722	257						
		604	fliY	b1920	800	603						
		298	fliT	b1926	365	297						
		238	amyA	b1927	1487	237						
		227	-	b1936	278	226						
		227	fliE	b1937	314	226						
		126	fliH	b1940	707	125						
		601	fliI	b1941	1373	600						
		286	fliN	b1946	413	285						
		442	fliP	b1948	737	441						
		388	fliR	b1950	785	387						
		574	rcsA	b1951	623	573						
		302	yedP	b1955	815	301						
		415	yedQ	b1956	1709	414						
		715	yodC	b1957	182	714						
		4	yedA	b1959	920	3						
		460	dcm	b1961	1418	459						
		361	yedU	b1967	851	360						
		253	yedV	b1968	1358	252						
		298	yedX	b1970	413	297						
		668	yedY	b1971	1004	667						
		98	yedZ	b1972	635	97						
		259	yodA	b1973	650	258						
		490	yeeI	b1976	836	489						
		292	amn	b1982	1454	291						
		760	yeeN	b1983	716	759						
		509	yeeO	b1985	1643	508						
		691	cbl	b1987	950	690						
		691	nac	b1988	917	690						
		272	erfK	b1990	932	271						

		738	yeeF	b2014	1364	737						
		738	yeeY	b2015	950	737						
		624	hisG	b2019	899	623						
		624	hisD	b2020	1304	623						
		76	hisB	b2022	1070	75						
		896	hisH	b2023	590	895						
		896	hisA	b2024	740	895						
		312	hisF	b2025	776	311						
		312	hisI	b2026	611	311						
		85	ugd	b2028	1166	84						
2111510	2112312	803	galF	b2042	893	802	Insd	KS	66	188	2111442	2112312
2126080	2126932	853	gmd	b2053	1121	852	OvrR	KS	50	216	2125960	2126932
			wcaF	b2054	548	852	Span					
			wcaE	b2055	746	852	OvrL					
2147533	2148214	682	yegI	b2070	1946	681	Insd	KS	138	313	2146783	2148583
2150349	2150378	30	yegJ	b2071/2 ?	761	29	Insd	OS	93	126	2150058	2150478
2154140	2154773	634	yegN	b2075	3122	633	Insd	KS	168	242	2153474	2155157
2156201	2156636	436			3122	435	OvrR	OS	65	105	2156201	2156636
			yegO	b2076	3077	435	OvrL					
2157620	2157848	229			3077	228	Insd	KO	43	145	2157566	2157962
2171298	2171415	118	gatC	b2092	1355	117	Insd	OS	60	114	2171289	2171415
2200354	2200447	94	yehI	b2118	3632	93	Insd	OS	124	122	2200351	2200447
2208749	2208833	85	yehQ	b2122	1868	84	Insd	OS	41	112	2208617	2208833
2245426	2246549	1124	lysP	b2156	1469	1123	Insd	KO	125	183	2244848	2249640
2265284	2268044	2761	yeiQ	b2172	1466	2760	OvrR	KS	152	174	2265284	
			yeiR	b2173	986	2760	Span					
			yeiU	b2174	749	2760	Span					
			spr	b2175	566	2760	OvrL					2268044
2277116	2277272	157	bcr	b2182	1190	156	Insd	KS	142	235	2276957	2278178
2339136	2339251	116	yfaL	b2233	3752	115	Insd	KO	128	127	2338626	2339374
2342059	2342242	184			3752	183	OvrR	OS	94	106	2342059	2342242
2371814	2371915	102	menE	b2260	1355	101	Insd	KO	175	247	2371777	
2373064	2373186	123	menC	b2261	962	122	Insd					2373225
2375685	2376528	844	menD	b2264	1670	843	Insd	KS	270	449	2373675	
2376792	2377474	683	menF	b2265	1070	682	OvrL					2379098

2396135	2396360	226	nuoG	b2283	2732	225	Insd		OS	56	119	2396135	2396387
2426850	2427804	955	purF	b2312	1517	954	Insd		KS	70	228	2426543	2427981
2440958	2441323	366	-	b2324	2066	365	Insd		KS	440	246	2440636	2441767
2482733	2483117	385	evgS	b2370	3593	384	Insd		KS	634	820	2482733	
2483545	2484134	590											
2484419	2484524	106											
2488826	2490805	1980	yfdU	b2373	1694	1979	OvrR						
			yfdW	b2374	1250	1979	OvrL						2492111
2496433	2496538	106							KO	40	121	2496433	2496588
2497381	2498754	1374	ypdA	b2380	1697	1373	OvrR		KS	317	276	2497381	2501661
			ypdB	b2381	734	1373	OvrL						
2500257	2501661	1405	ypdD	b2383	2495	1404	Insd						
2517242	2518412	1171	gltX	b2400	1415	1170	OvrL	***	KS	53	216	2517242	2518802
2538203	2538290	88	cysA	b2422	1097	87	Insd		KO	113	102	2538203	2538290
2539365	2540012	648	cysU	b2424	833	647	OvrL		KS	104	174	2539365	2540012
2549048	2549547	500	yfeY	b2432	575	499	OvrR		KO	92	113	2548971	2549547
			yfeZ	b2433	455	499	OvrL						
2577878	2578703	826	tktB	b2465	2003	825	Insd		KS	94	208	2577878	2579387
2631069	2631372	304	guaB	b2508	1466	303	Insd		OS	164	126	2630826	2631426
2656345	2656738	394	hscA	b2526	1850	393	Insd		KO	78	119	2656345	2657261
2673624	2674019	396	yphC	b2545	1094	395	OvrR		KS	198	210	2673624	2674181
			yphD	b2546	998	395	OvrL						
2688733	2689535	803	yfhK	b2556	1490	802	OvrR		OS	49	106	2688733	2689535
			??	b4441	148	802	Span						
2695690	2696331	642	tadA	b2559	536	641	OvrR		KS	70	227	2695681	2697520
			yfhB	b2560	572	641	OvrL		KS	319	645	2697876	2698684
2698155	2698552	398											
2699661	2700517	857	pdxJ	b2564	731	856	OvrR		KO	127	113	2699661	2700889
			recO	b2565	728	856	Span						
			era	b2566	905	856	OvrL						
2709565	2710024	460	nadB	b2574	1622	459	Insd		KO	71	134	2708886	2710024
2710558	2711627	1070	yfiC	b2575	857	1069	OvrR		OS	70	102	2710558	2711627
			srmB	b2576	1334	1069	OvrL						
2792079	2792594	516	gabP	b2663	1400	515	OvrL		KO	165	179	2791841	2792630
2800301	2800970	670		b2675	2144	669	Insd		KO	124	124	2800118	2800970
2806623	2808012	1390	-	b2681	917	1389	OvrR		OS	88	105	2806623	2808012

			ygaZ	b2682	737	1389	OvrL						
2816836	2817920	1085	csrA	b2696	185	1084	Span		OS	74	138	2816836	2818296
			alaS	b2697	2630	1084	OvrL	***					
2822179	2822734	556	ygaD	b2700	497	555	OvrR		KS	333	230	2822179	2826441
			mltB	b2701	1085	555	OvrL						
2829735	2830215	481	ygaA	b2709	1589	480	Insd		KS	68	181	2829735	2830215
2855806	2856025	220	mutS	b2733	2561	219	Insd		KO	92	176	2855665	2856025
2857015	2857216	202			2561	201	Insd		KS	188	229	2856172	2857606
2857996	2858044	49	pphB	b2734	656	48	Insd		OS	42	115	2857995	2858044
2885479	2886097	619	cysH	b2762	734	618	OvrL		KO	125	133	2885477	2886133
2887764	2887877	114	cysI	b2763	1712	113	Insd		OS	61	118	2887518	2887877
2892468	2892897	430	ygcP	b2768	575	429	OvrR		OS	94	115	2892468	2892949
2955379	2955757	379	ptr	b2821	2888	378	Insd		KS	216	243	2955379	
2959033	2959700	668	recC	b2822	3368	667	Insd						2956039
3037483	3040087	2605	xerD	b2894	896	2604	OvrR		KS	95	197	3037483	
			fldB	b2895	521	2604	Span						
			ygfX	b2896	407	2604	Span						
			ygfY	b2897	266	2604	Span						
			ygfZ	b2898	980	2604	OvrL						3040290
3054888	3055427	540		b4446	150	539	OvrR		OS	67	111	3054888	
			serA	b2913	1232	539	OvrL						3055718
3065823	3066533	711	yggE	b2922	740	710	OvrR		OS	68	260	3065472	
			yggA	b2923	635	710	OvrL						
3066623	3066920	298											3067574
3078508	3078811	304	tktA	b2935	1991	303	Insd		OS	85	106	3078508	3078811
3086049	3086697	649	galP	b2943	1394	648	OvrL		KO	70	115	3086049	3086697
3092781	3092871	91	yggR	b2950	1025	90	Insd		KO	58	143	3092781	3092871
3107479	3107525	47							OS	30	103	3107479	3107525
3150361	3150535	175	metC	b3008	1187	174	Insd		OS	128	157	3150361	3151435
3157365	3157686	322	ygiQ	b4469	2219	321	Insd		OS	82	133	3157365	3157686
3173190	3173881	692	parE	b3030	1892	691	OvrR		KO	66	117	3173064	
			yqiA	b3031	581	691	OvrL						3173881
3193548	3193680	133	rfaE	b3052	1433	132	Insd		KO	101	104	3193548	3193761
3195707	3195878	172	glnE	b3053	2840	171	Insd		KO	183	195	3195647	3196208
3217880	3218417	538	ygjG	b3073	1490	537	Insd		OS	125	127	3217859	3218417
3222285	3222879	595	ebgA	b3076	3092	594	Insd		KS	130	225	3221757	3222879

3265686	3265744	59							KO	27	128	3265686	3265744
3302942	3304043	1102	mtr	b3161	1244	1101	OvrR		KO	108	105	3302942	
			deaD	b3162	1940	1101	OvrL						3304043
3320864	3320979	116	glmM	b3176	1337	115	Insd		KS	180	263	3320864	3324171
3355874	3356426	553	gltB	b3212	4553	552	Insd		KO	161	118	3355442	3356426
3419785	3420597	813	yhdY	b3270	1106	812	OvrR		KO	65	101	3419785	
			yhdZ	b3271	758	812	OvrL						3420597
3430138	3430351	214	smg	b3284	473	213	Insd		KS	180	317	3426691	3430672
3493780	3494548	769	nirB	b3365	2543	768	Insd		KO	138	146	3493780	3495194
3519180	3519937	758	yrfB	b3393	440	757	OvrR		KS	435	319	3510784	
			yrfC	b3394	539	757	OvrL						3519937
3536917	3537593	677	yhgF	b3407	2222	676	Insd		OS	63	124	3536635	3537754
3552671	3552839	169	malT	b3418	2705	168	Insd		OS	66	107	3552671	3553193
3561021	3561282	262	glpD	b3426	1505	261	Insd		KO	57	109	3561021	3561282
3584065	3584693	629	ggt	b3447	1742	628	Insd		KS	65	202	3583883	
3606869	3607457	589	sirA	b3470	245	588	OvrR						3584747
			yhhQ	b3471	665	588	OvrL		KS	166	320	3606765	
3614669	3615363	695	nikC	b3478	833	694	OvrR						3609546
			nikD	b3479	764	694	OvrL		KS	206	239	3614383	3615911
3679057	3684376	5320	yhjJ	b3527	1496	5319	OvrR		KS	190	173	3679057	
			dctA	b3528	1286	5319	Span						
			yhjK	b3529	1955	5319	Span						
			yhjL	b3530	3422	5319	OvrL						3684832
3721289	3721394	106	glyS	b3559	2069	105	Insd	***	OS	143	179	3720653	3721478
3732275	3732797	523	xylH	b3568	1181	522	Insd		KS	76	226	3732054	3733837
3755843	3756747	905	yiaY	b3589	1151	904	OvrR		OS	77	120	3755843	3756747
3773387	3773434	48	selB	b3590	1844	904	OvrL		OS	75	171	3773246	3773434
3779988	3780132	145	mtlD	b3600	1148	47	Insd		OS	309	189	3779683	
3783097	3783935	839	cysE	b3607	821	144	Insd						
3789683	3790136	454	yibO	b3612	1544	838	OvrL						3784997
3815811	3815844	34	kbl	b3617	1196	453	Insd		KO	53	120	3789683	3790202
3818503	3818564	62	dinD	b3645	836	33	Insd		KS	29	350	3815811	3815866
3824658	3824941	284	yicF	b3647	1688	61	Insd		KO	107	114	3817312	3818564
3824974	3825142	169	recG	b3652	2081	283	Insd		OS	33	116	3824545	3824941
				b3652	2081	168	Insd		KS	24	234	3824974	3825142
3831783	3833355	1573	yicI	b3656	2318	1572	OvrR		KS	128	283	3830436	

			yicJ	b3657	1439	1572	OvrL						3833469
3838706	3838811	106	yicM	b3662	1355	105	Insd		KS	98	850	3838320	
3838852	3839027	176											
3839028	3839217	190											3839217
3843629	3845969	2341	ade	b3665	1766	2340	OvrR		KS	263	258	3843581	
			uhpT	b3666	1391	2340	Span						
			uhpC	b3667	1322	2340	OvrL						
3847732	3848347	616	uhpB	b3668	1505	615	OvrR						
			uhpA	b3669	590	615	OvrL						3848524
3877044	3877740	697	gyrB	b3699	2414	696	Insd		OS	91	111	3877044	3878497
3880846	3885329	4484	dnaA	b3702	1403	4483	OvrR		KS	164	175	3880846	
			rpmH	b3703	140	4483	Span	**!!!!**					
			rnpA	b3704	359	4483	Span						
			yidC	b3705	1646	4483	Span						
			trmE	b3706	1364	4483	OvrL						3885329
3894599	3894803	205	yieG	b3714	1337	204	OvrR		KS	34	429	3894599	
			yieH	b3715	665	204	OvrL						3894803
3895149	3895302	154							KS	42	371	3895149	3895356
3952894	3952933	40	ilvD	b3771	1850	39	Insd		KO	121	141	3952894	3952963
3958842	3958957	116	rep	b3778	2021	115	Insd		KO	99	179	3958842	3959100
3984954	3985278	325	hemY	b3802	1196	324	Insd		KO	48	119	3984900	3985278
4027265	4027811	547	fadB	b3846	2189	546	Insd		KS	174	201	4026849	4027811
4037408	4038340	933	pepQ	b3847	2904	932	Insd		OS	32	103	4037408	4038340
4039728	4040166	439	mobA	b3857	584	438	OvrR		OS	120	144	4039728	
4078354	4078696	343	yihD	b3858	269	438	OvrL						4041115
			fdhE	b3891	929	342	Insd		KO	59	116	4078354	4078762
4079972	4081097	1126	fdoH	b3893	902	1125	OvrR		KS	139	177	4079972	
			fdoG	b3894	3050	1125	OvrL						4081097
4081766	4081985	220			3050	219	Insd		OS	54	101	4081766	4081985
4091938	4092043	106	rhaD	b3902	824	105	Insd		OS	62	257	4091893	
4092070	4092166	97											4092166
4093030	4093516	487	rhaA	b3903	1259	486	Insd		KS	103	234	4093030	4093951
4094386	4094718	333	rhaB	b3904	1469	332	Insd		KO	174	161	4094265	4097028
4141768	4142728	961	frwB	b3950	320	960	OvrR		KS	174	255	4141520	
			pflD	b3951	2297	960	OvrL						4142728
4162435	4162700	266	btuB	b3966	1844	265	Insd		KS	102	196	4162188	4162700

4200742	4202298	1557	zraS	b4003	1397	1556	OvrR	KS	270	214	4200742	
			zraR	b4004	1325	1556	OvrL					
4203201	4203816	616	purD	b4005	1289	615	Insd					4203816
4281350	4281575	226	actP	b4067	1649	225	Insd	KO	47	104	4281350	4281575
4281860	4283077	1218		b4067	1649	1217	OvrR	KS	93	212	4281686	4283077
			yjcH	b4068	314	1217	OvrL	KS	125	199	4386769	
4386769	4387825	1057	yjeP	b4159	3323	1056	OvrR					
			psd	b4160	968	1056	OvrL					4388797
4416990	4417578	589	yjfR	b4192	1070	588	Insd	KO	135	132	4415186	4417578
4418313	4418403	91	sgaT	b4193	1454	90	Insd	KO	308	435	4417966	
4418487	4418718	232										
4419978	4420465	488	ptxA	b4195	464	487	OvrR					
			sgaH	b4196	650	487	OvrL					4420819
4430428	4430593	166	ytfF	b4210	974	165	Insd	KO	90	134	4430409	4431261
4449322	4449636	315	ytfR	b4485	1502	314	Insd	KO	116	131	4449322	4450053
4464876	4465744	869	treR	b4241	947	868	OvrR	KO	237	658	4464564	
			mgtA	b4242	2696	868	OvrL					
4465895	4466108	214										
4466114	4466628	515										
4466867	4468061	1195										
4468163	4468368	206										
4469338	4469896	559	pyrI	b4244	461	558	OvrR					
			pyrB	b4245	935	558	OvrL					4469896
4486901	4487030	130	yjgR	b4263	1502	129	Insd	KO	118	130	4486643	4487030
4538498	4538826	329						KO	90	106	4538498	4538826
4623864	4624660	797	serB	b4388	968	796	OvrR	KO	69	115	4623864	
			sms	b4389	1382	796	OvrL					4624695
4626218	4626503	286						KO	69	187	4625456	4626503

total MSC length 128515
